# Supplementary material for: A Past Genetic Bottleneck from Argentine Beans and a Selective Sweep Led to the Race Chile of the Common Bean (Phaseolus vulgaris L.)
Source: Int J Mol Sci. 2024 Apr 6;25(7):4081. doi: 10.3390/ijms25074081 (PMC11012279; doi:10.3390/ijms25074081)
Supplement: Supplementary file 1 [file ijms-25-04081-s001.zip › ijms-2948514-supplementary/Supplementary figures/Figure S4.pdf]

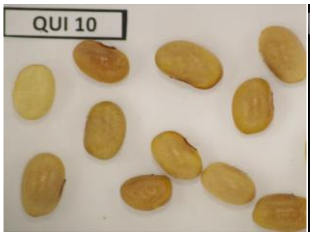

"Burro Argentino"

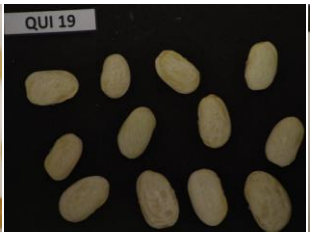

"Coscorron Corriente"

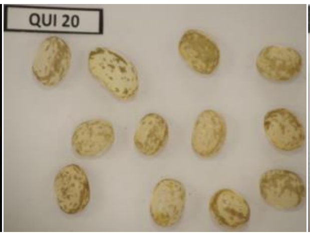

"Hallado"

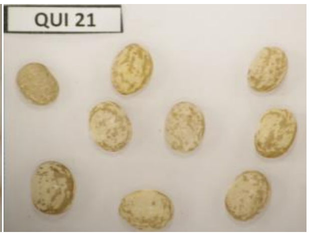

"Hallado Chico"

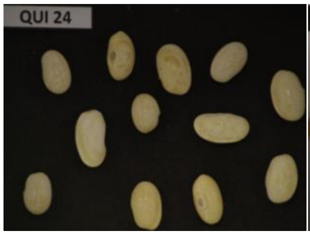

"Coscorron Mendez 1"

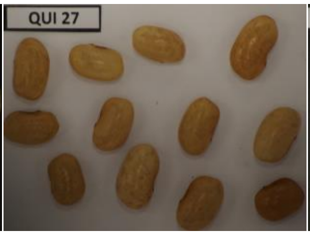

"Bayas"

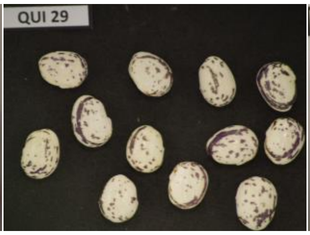

"Sapito"

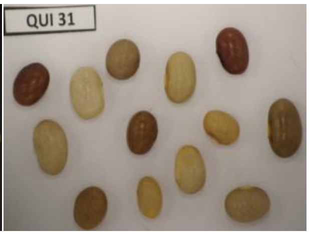

"Burro"

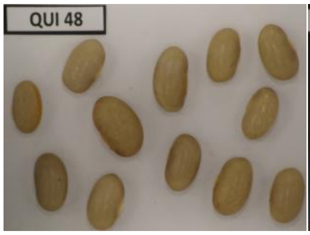

"Tortola Corriente"

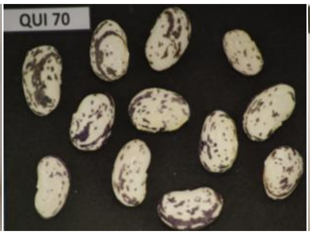

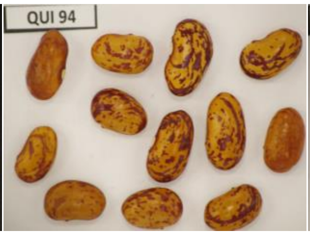

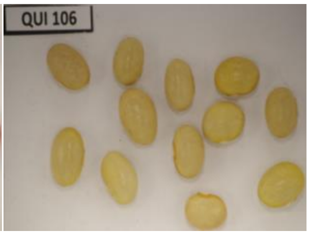

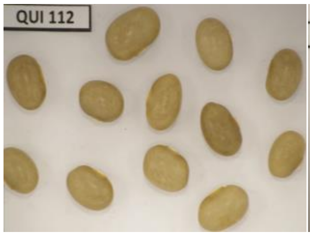

"Tortola"

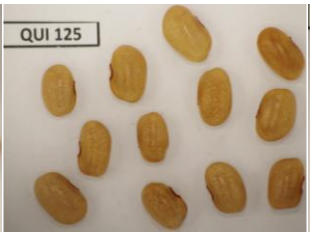

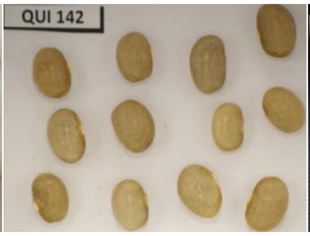

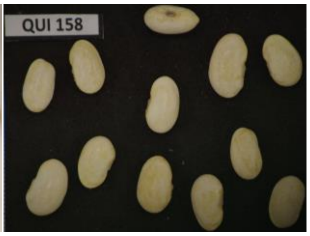

"Coscorron"

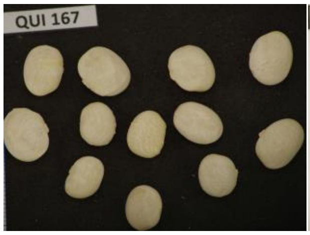

"Pajarito"

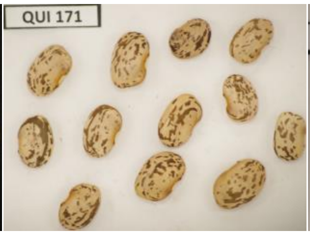

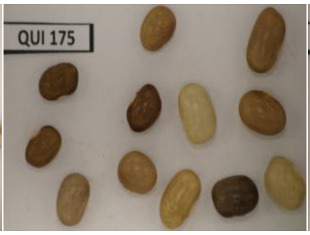

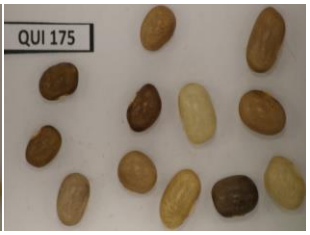

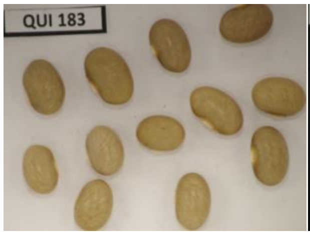

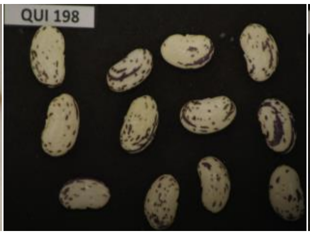

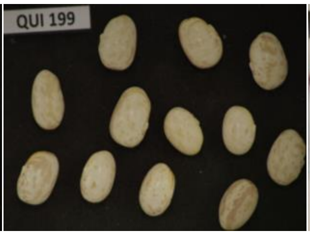

"Coscorron"

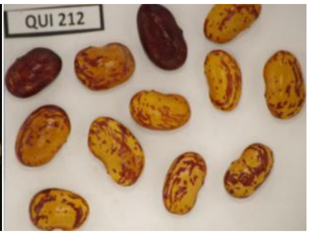

"Bio-Bio"

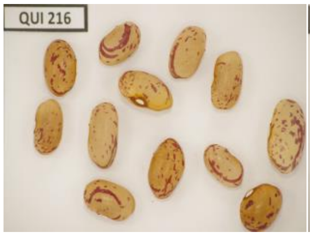

“Frutilla”

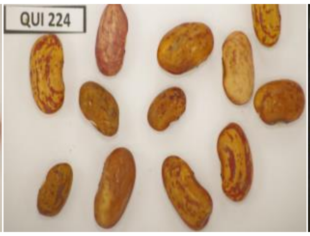

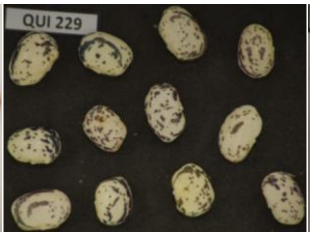

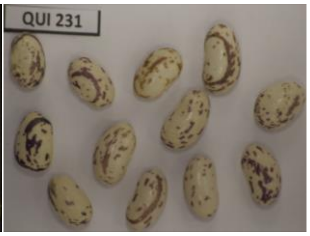

“Sapito”

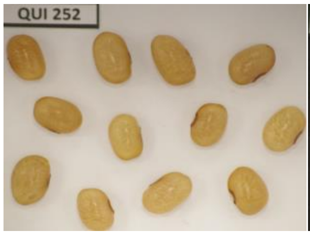

“Burro Argentino”

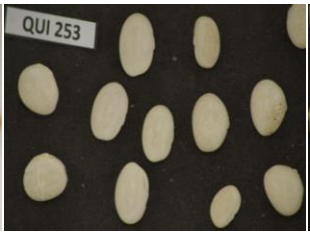

“Mantequilla”

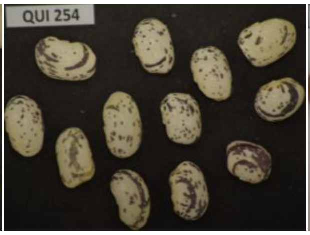

“Pajarito”

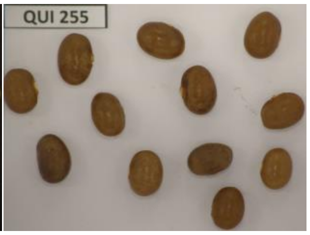

“Burro Chileno”

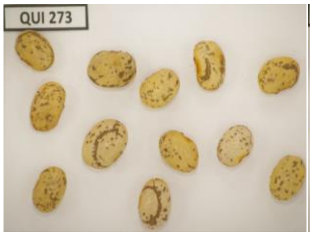

“Diuca”

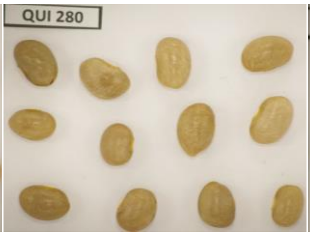

“Burrito Argentino”

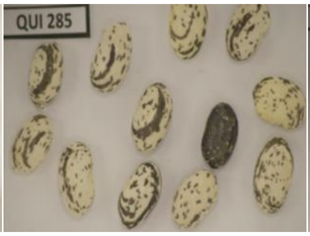

“Hallado Chileno”

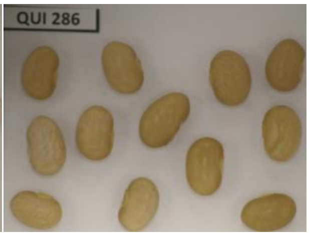

“Tortola Corriente”

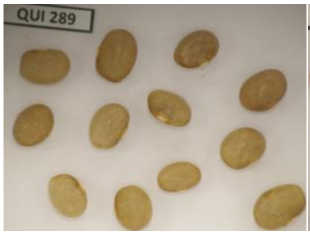

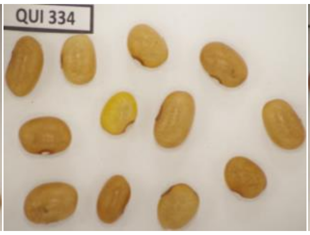

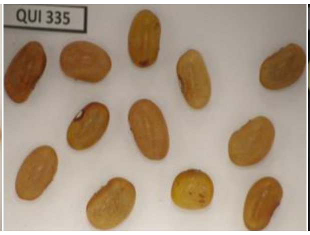

“Siete Semanas”

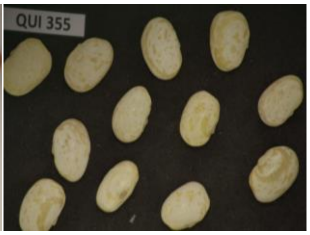

“Coscorron”

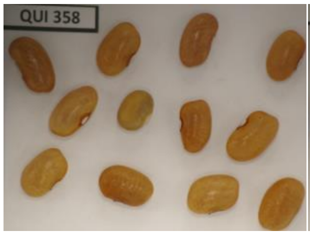

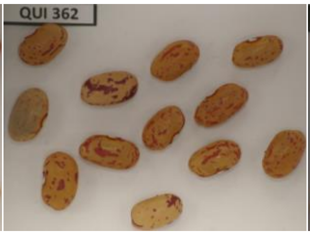

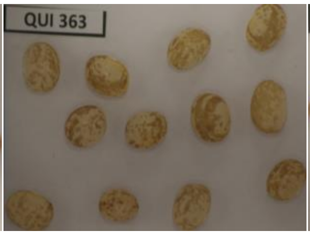

“Hallado Chico”

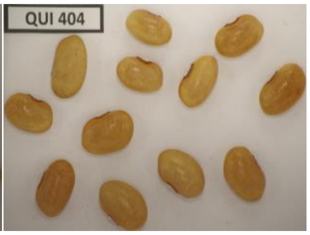

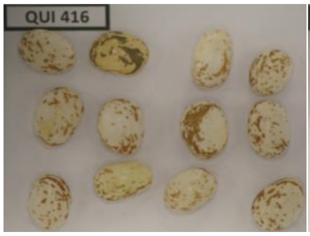

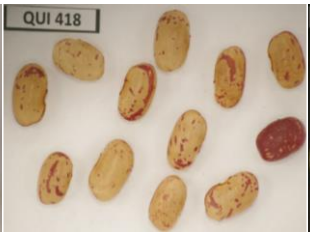

“Peumo”

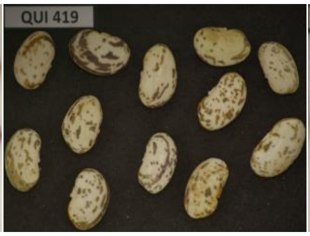

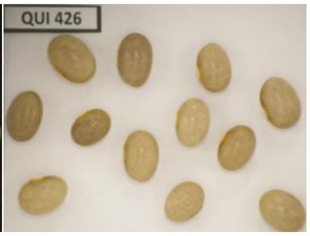

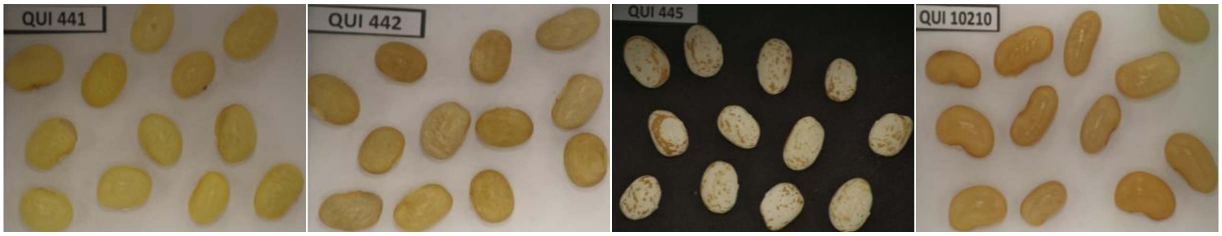

“Tortola”

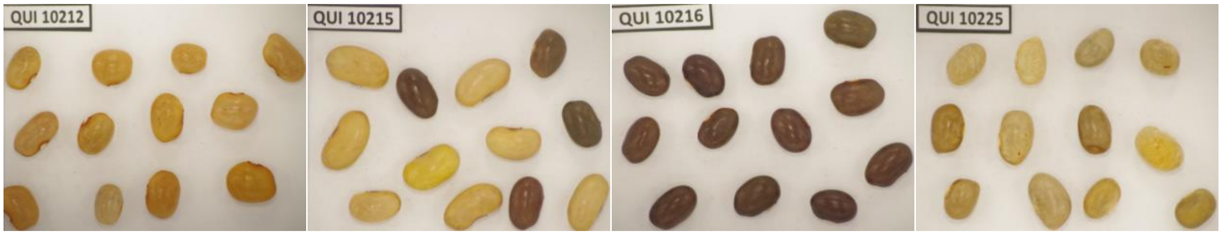

“Rancaguino” “Burritos”

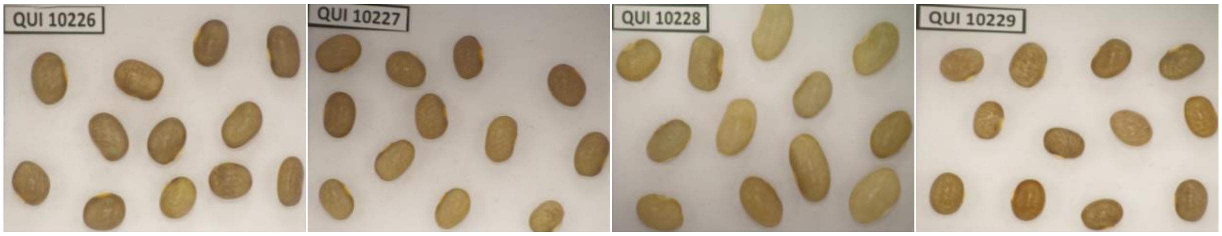

“Plomo” “Plomo 100 Dias” “Burros Antiguos”

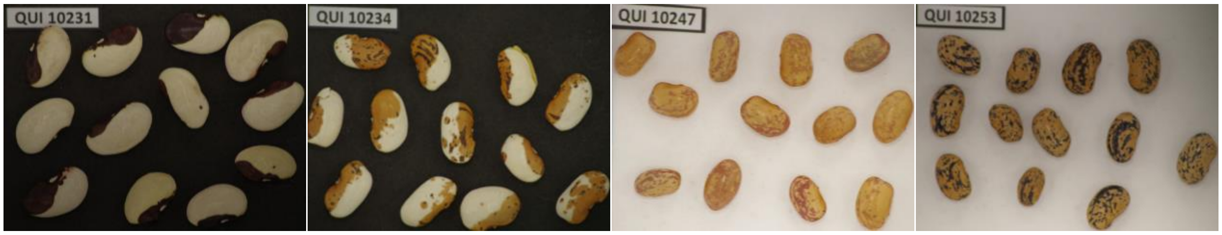

“Ojo Negro” “Gato” “Frutilla” “Chilotito”

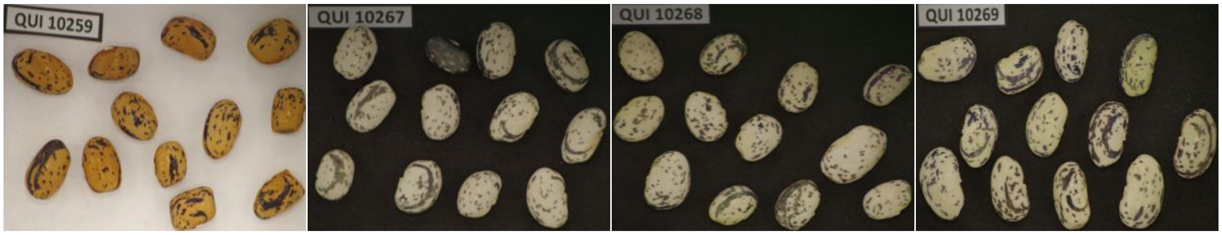

“Sapito”

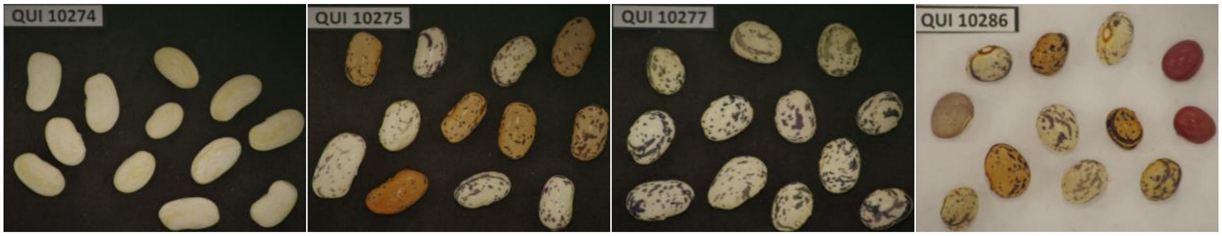

“Sapito” “Sapo” “Sapito”

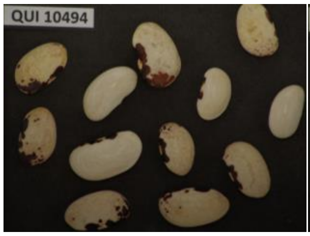
QUI 10494
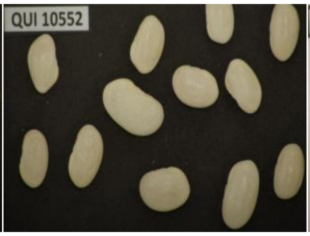
QUI 10552
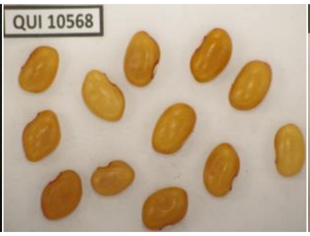
QUI 10568
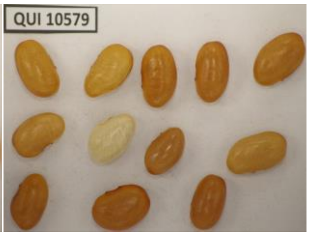
QUI 10579

"Cabrito"

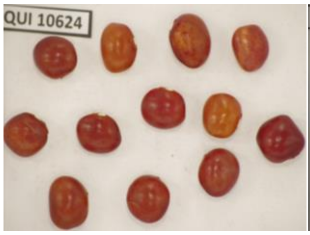
QUI 10624
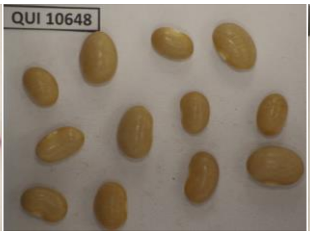
QUI 10648
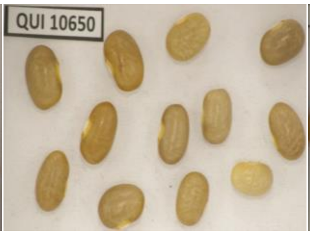
QUI 10650
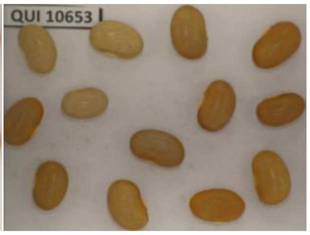
QUI 10653
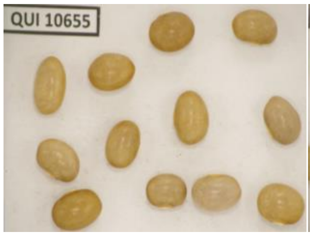
QUI 10655
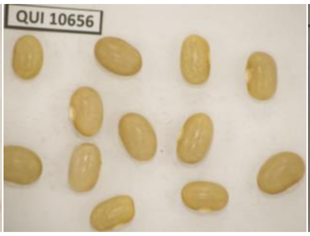
QUI 10656
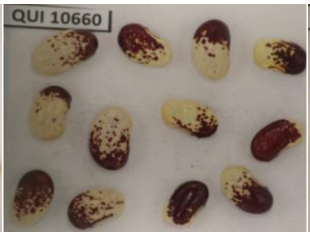
QUI 10660
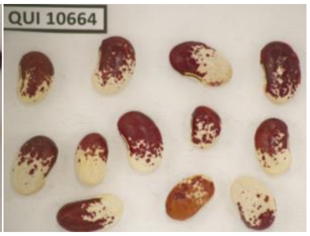
QUI 10664
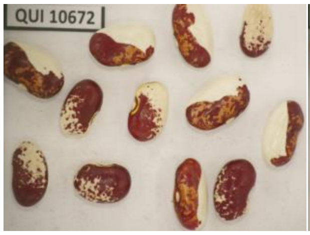
QUI 10672
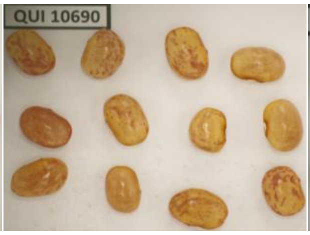
QUI 10690
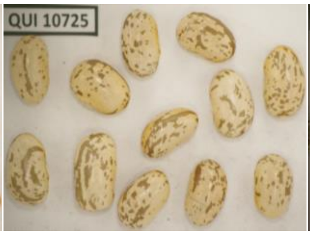
QUI 10725
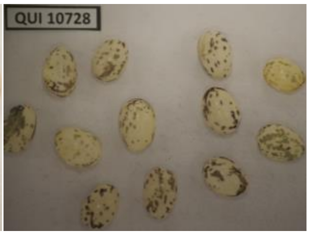
QUI 10728
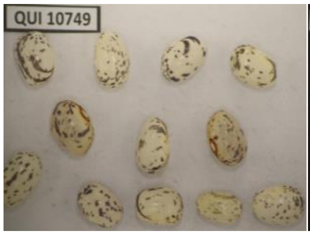
QUI 10749
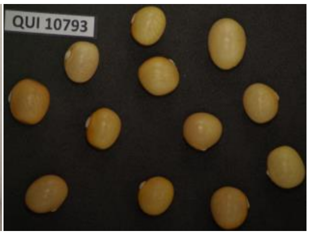
QUI 10793
