# Supplementary figures and images for: A Past Genetic Bottleneck from Argentine Beans and a Selective Sweep Led to the Race Chile of the Common Bean (Phaseolus vulgaris L.)
Source: Int J Mol Sci. 2024 Apr 6;25(7):4081. doi: 10.3390/ijms25074081 (PMC11012279; doi:10.3390/ijms25074081)

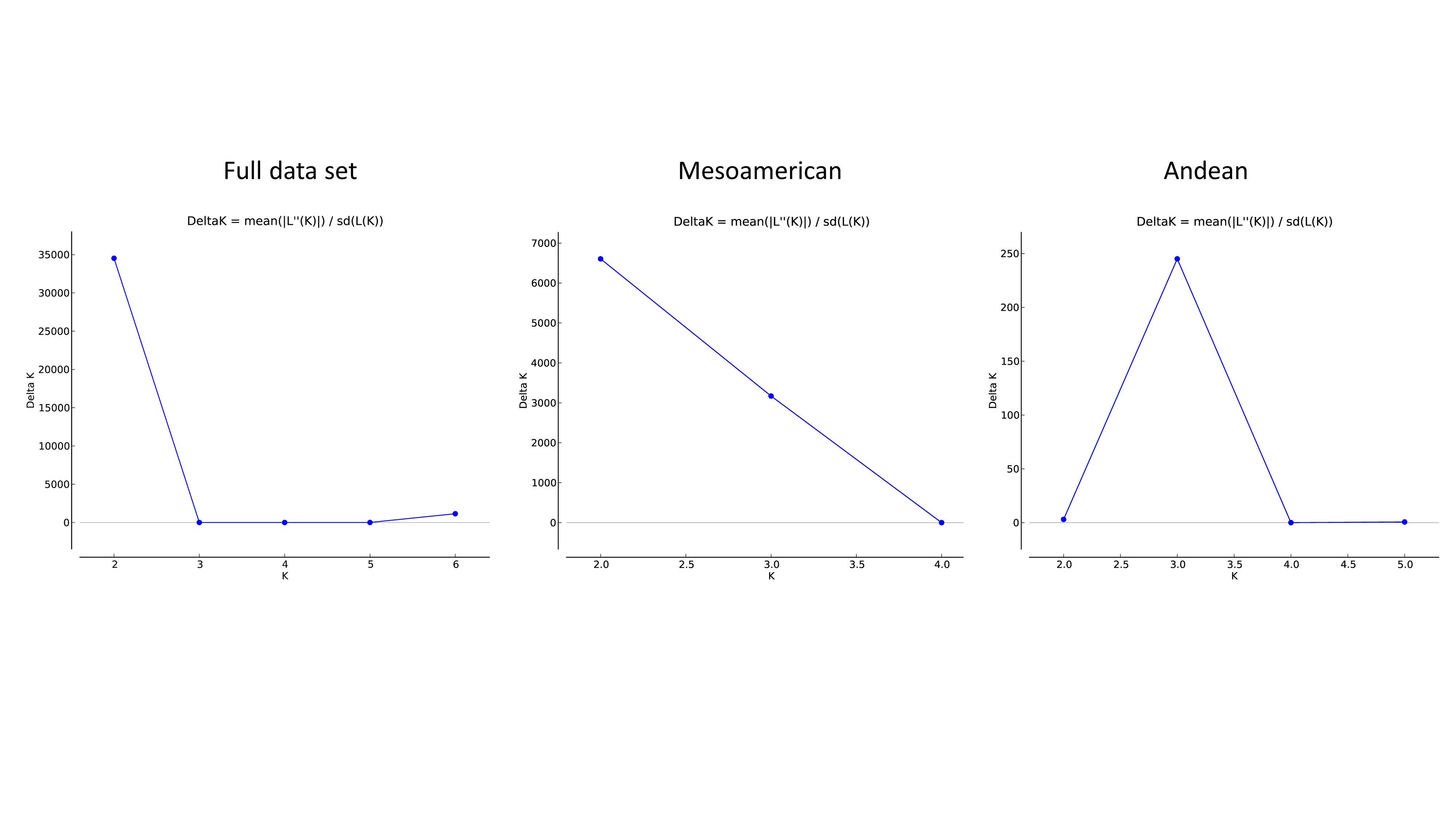

Supplement: Supplementary file 1 [file ijms-25-04081-s001.zip › ijms-2948514-supplementary/Supplementary figures/Figure S2.TIFF]

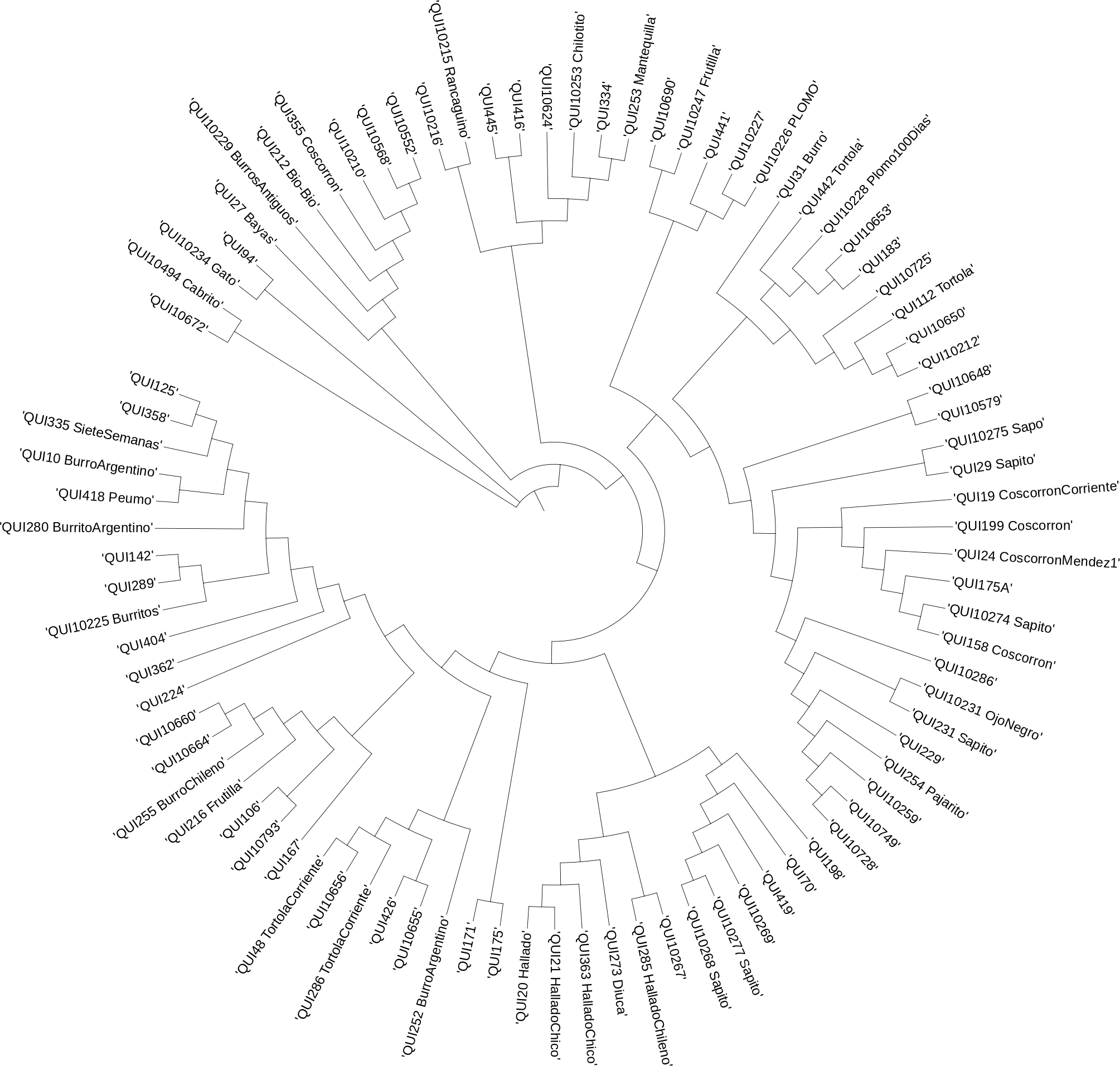

Supplement: Supplementary file 1 [file ijms-25-04081-s001.zip › ijms-2948514-supplementary/Supplementary figures/Figure S3.TIFF]

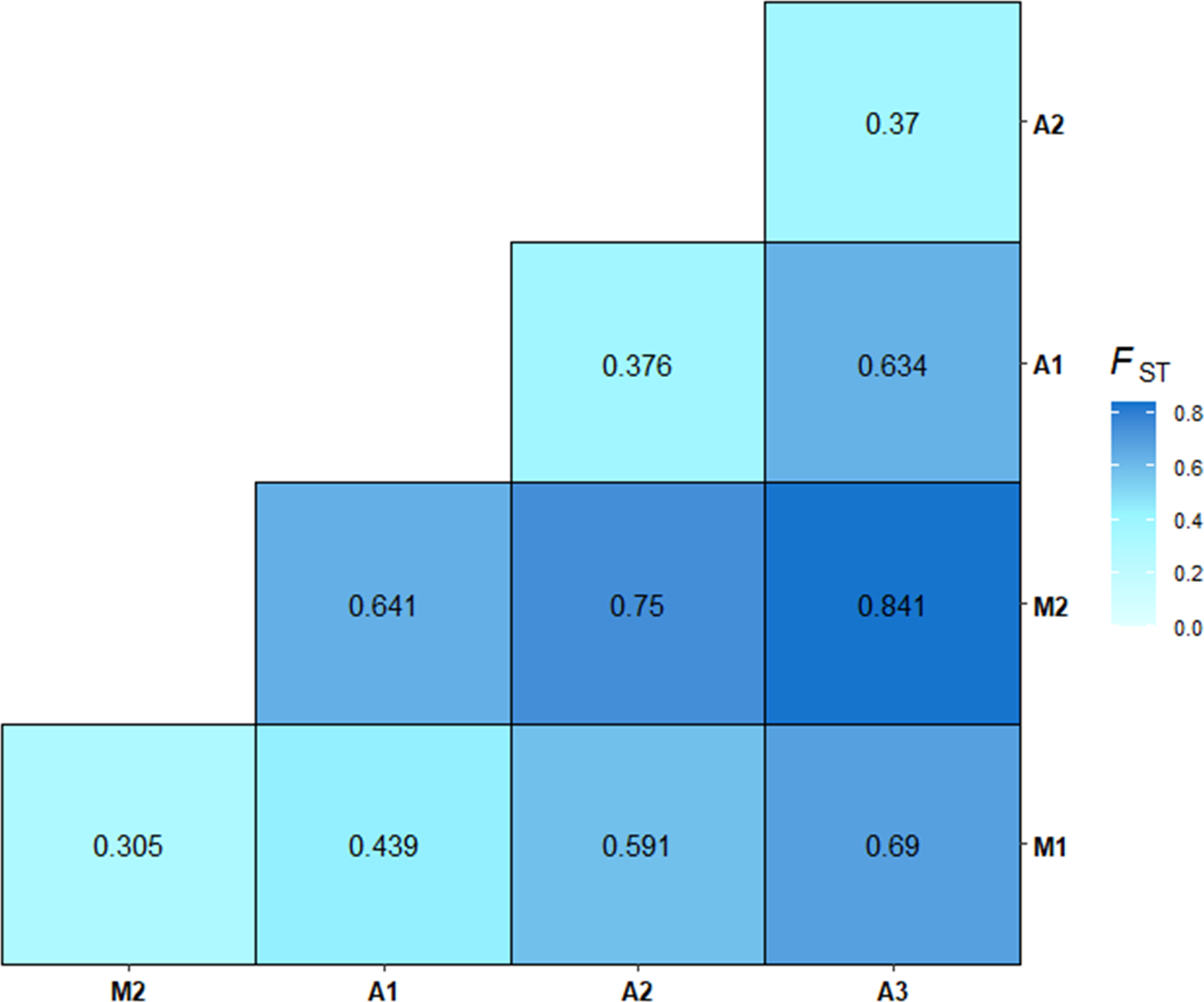

Supplement: Supplementary file 1 [file ijms-25-04081-s001.zip › ijms-2948514-supplementary/Supplementary figures/Figure S5.TIFF]

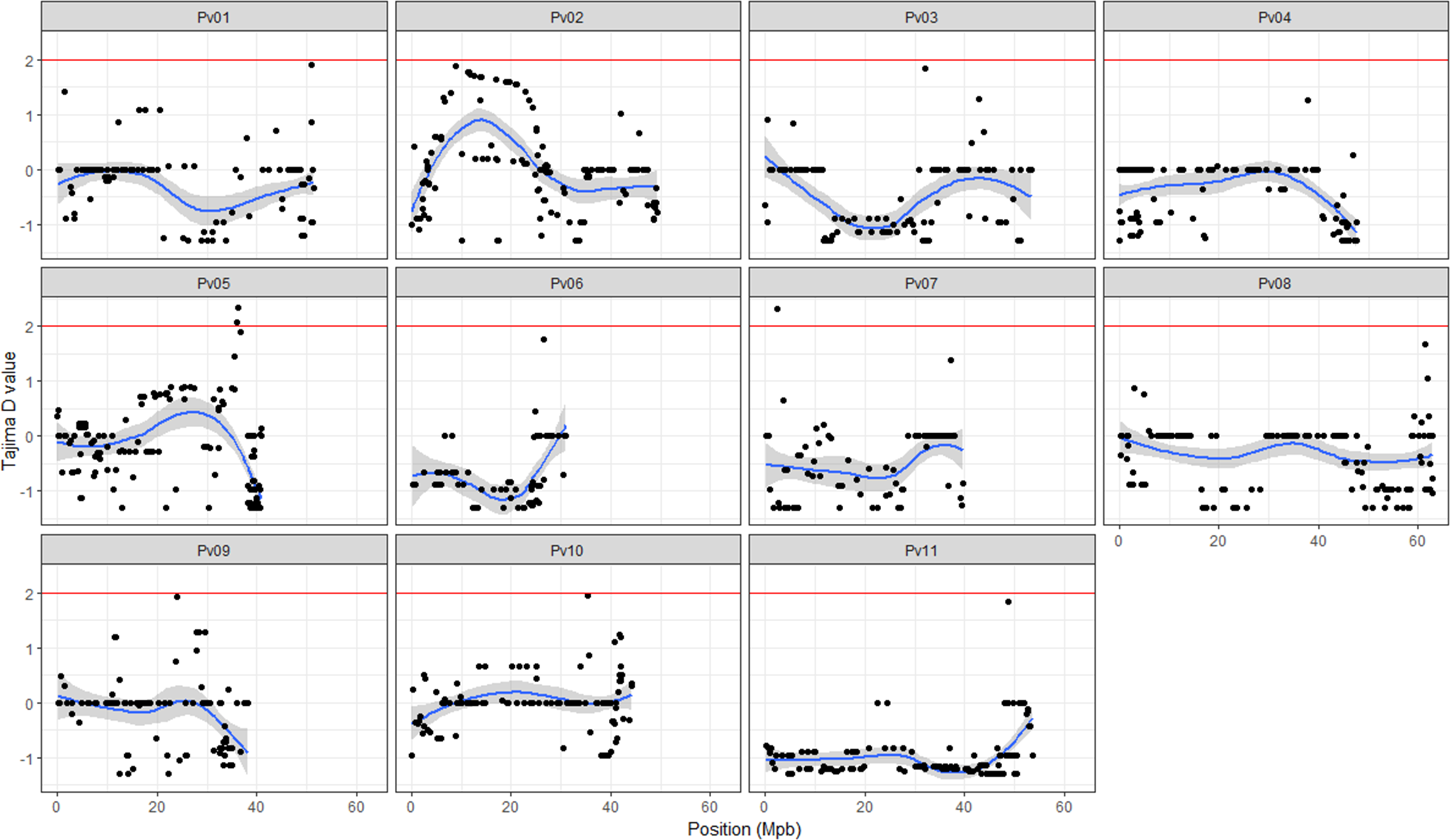

Supplement: Supplementary file 1 [file ijms-25-04081-s001.zip › ijms-2948514-supplementary/Supplementary figures/Figure S6.TIFF]

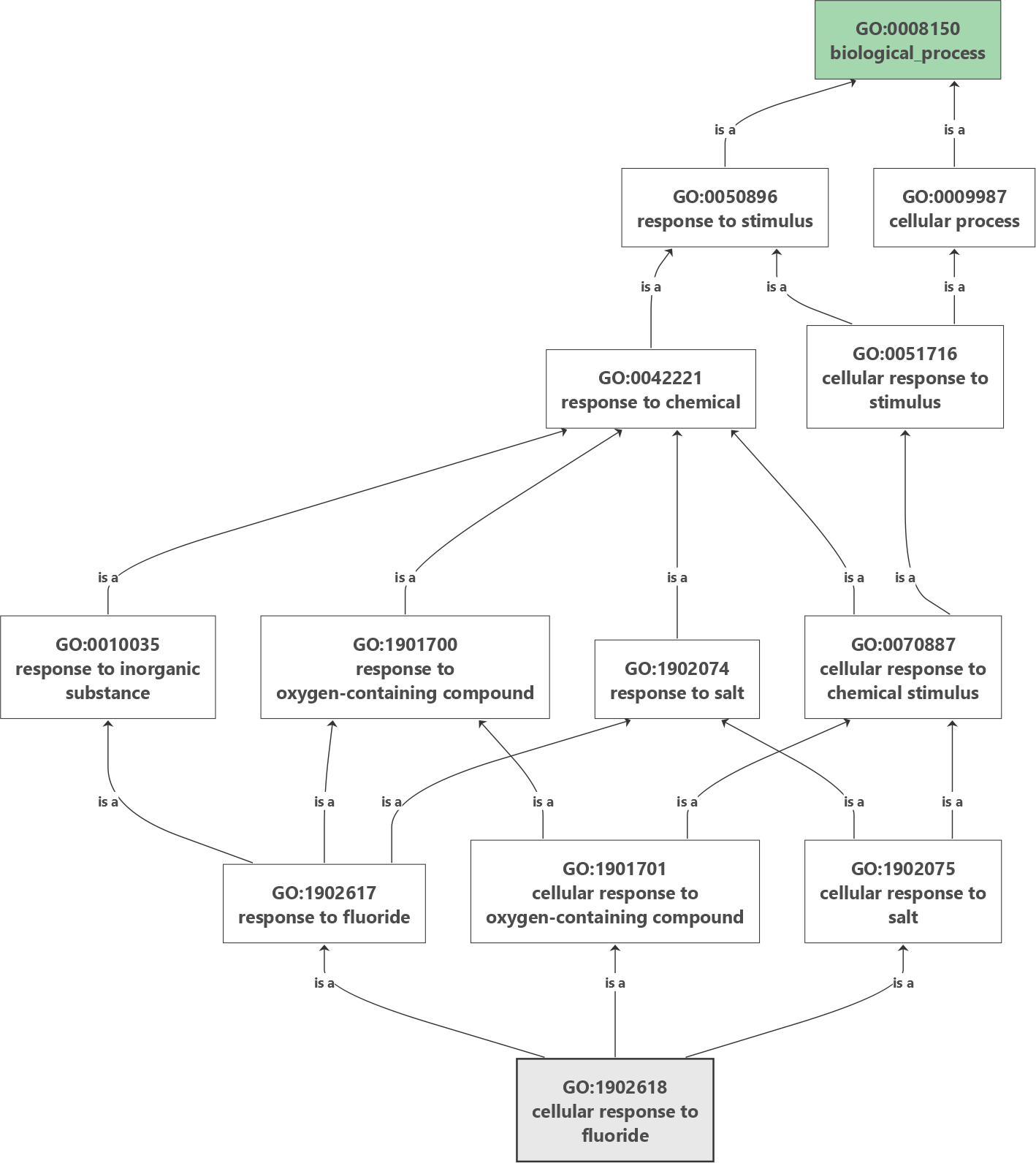

Supplement: Supplementary file 1 [file ijms-25-04081-s001.zip › ijms-2948514-supplementary/Supplementary figures/Figure S7.TIFF]
